# Improving the Ionic Conductivity of PEGDMA-Based Polymer Electrolytes by Reducing the Interfacial Resistance for LIBs

**DOI:** 10.3390/polym14173443

**Published:** 2022-08-23

**Authors:** Lei Jin, Giseok Jang, Hyunmin Lim, Wei Zhang, Sungjun Park, Minhyuk Jeon, Hohyoun Jang, Whangi Kim

**Affiliations:** Department of Applied Chemistry, Konkuk University, 268 Chungwon-daero, Chungju-si, Seoul 27478, Chungcheongbuk-do, Korea

**Keywords:** polymer electrolyte, lithium-ion batteries, interfacial resistance, in situ, low-molecular-weight PEGDMA

## Abstract

Polymer electrolytes (PEs) based on poly(ethylene oxide) (PEO) have gained increasing interest in lithium-ion batteries (LIBs) and are expected to solve the safety issue of commercial liquid electrolytes due to their excellent thermal and mechanical stability, suppression of lithium dendrites and shortened battery assembly process. However, challenges, such as high interfacial resistance between electrolyte and electrodes and poor ionic conductivity (σ) at room temperature (RT), still limit the use of PEO-based PEs. In this work, an in situ PEO-based polymer electrolyte consisting of polyethylene glycol dimethacrylate (PEGDMA) 1000, lithium bis(fluorosulfonyl)imide (LiFSI) and DMF is cured on a LiFePO_4_ (LFP) cathode to address the above-mentioned issues. As a result, optimized PE shows a promising σ and lithium-ion transference number (*t*_Li_^+^) of 6.13 × 10^−4^ S cm^−1^ and 0.63 at RT and excellent thermal stability up to 136 °C. Moreover, the LiFePO_4_//Li cell assembled by in situ PE exhibits superior discharge capacity (141 mAh g^−1^) at 0.1 C, favorable Coulombic efficiency (97.6%) after 100 cycles and promising rate performance. This work contributes to modifying PEO-based PE to force the interfacial contact between the electrolyte and the electrode and to improve LIBs’ performance.

## 1. Introduction

Due to their high operating voltage, energy density, long (cycle) lifespan and low memory effect, LIBs have been widely used in electric vehicles, energy storage systems and various portable devices [1,2,3,4]. Electrolytes, as one of the most important components in LIBs, facilitate the movement of lithium ions during charging and discharging the battery [5,6,7]. Nowadays, a liquid electrolyte consisting of 1 M lithium hexafluorophosphate (LiPF_6_) in a mixture of ethylene carbonate and linear carbonate solvents is used as a commercial material in LIBs because of its high ionic conductivity (10^−2^ S cm^−1^) and cyclical stability at RT [8,9,10]. However, the flammability, volatilization and leakage of organic solvents at high temperatures and operating voltage also increase the safety concerns for the liquid electrolyte [11,12,13]. Moreover, the PF_6_^−^ anion reacts with trace amounts of impurities, moisture and alcohol to release gaseous HF, which has a detrimental effect on the electrodes and the cyclical performance of batteries [14,15,16,17]. To address safety issues, more and more attention is being paid to solid electrolytes due to the absence of flammable organic solvents, which significantly alleviates the safety concerns regarding the electrolyte [18,19,20,21,22]. Inorganic solid electrolytes provide high σ and outstanding electrochemical stability at RT, which can also suppress lithium dendrites and maintain excellent thermal stability [23,24,25]. However, practical commercial application is restricted by the expensive raw material and the difficult sintering process to achieve close interfacial contact with the electrodes [26,27]. In terms of organic electrolytes, poly (acrylonitrile) (PAN), poly (methyl methacrylate) (PMMA) and poly (vinylidene fluoride-hexafluoro propylene) (PVdF-HFP), etc., have been widely studied and reported as the polymeric matrix over the past century [28,29,30,31]. Recently, researchers have also shown an increased interest in imidazolium, amine, silicon, epoxy, urethane and dextrin functionalized monomers to modify polymer electrolytes to meet the requirements of high-performance LIBs [32,33,34,35,36,37]. However, extensive research is still focused on PEO-based solid polymer electrolytes, because its high dielectric constant provides sufficient solubility in lithium salt and a high donor number for lithium ions [38,39]. In PEO-based PEs, the electro-donating group of ethylene oxide can coordinate with lithium ions and ion transport is conducted by a lithium–oxygen bond breaking and forming in the polymer electrolyte [40,41]. Nevertheless, the advantage is offset by the low σ (10^−6^ S cm^−1^) at RT due to the large crystalline volume of PEO and the huge phase interfacial resistance between electrolyte and electrodes, resulting in more and more research focusing on PEO modification and trying to improve their RT σ [42,43]. J. Atik et al. [44] prepared pyrrolidinium cation ionic liquids as a plasticizer for PEO-based SPEs to lower the crystalline phase in the electrolyte and increase σ and lithium transport in lithium–metal polymer batteries. Fang et al. [45] also reported that an optimized amount of Li_2_S_6_ added to a PEO-based polymer electrolyte not only formed a stable Li/PEO interface, but also enhanced the σ to 1.7 × 10 ^−4^ S cm^−1^ at 40 °C. 

In this work, in order to further reduce the interfacial impedance and form an intimate connection between the electrode and the electrolyte, in situ UV-cured photopolymerization was utilized to prepare a polymer electrolyte on the LFP cathode. Our previous work [46] showed that this facile method could force electrode/electrolyte interfacial compatibility and radically reduce the interface resistance, while the assembled cells exhibited promising σ of 1.16 × 10^−4^ S cm^−1^ and a good *t*_Li_^+^ of 0.61 at RT and significantly better than the ex situ formed counterpart. Besides, we attempted to synthesize low-molecular-weight PEGDMA (average Mw = 1000) as a polymer matrix because low-molecular-weight PEO easily contributed to a higher σ than high-molecular-weight polymers under the same conditions [47]. Moreover, DMF was added as an additive to the electrolyte to improve the electrolyte performance. Recent research from You et al. [48] demonstrated that adding DMF to a liquid electrolyte could snatch at the PF_5_ from LiPF_6_ decomposition and form a uniform cathode electrolyte interface (CEI) on the LFP, which prevents the LFP cathode from depleting during battery operation and improves LIBs’ performance at high temperature (60 °C). As a result, the optimized prepared PE revealed satisfactory thermal stability (136 °C), high σ 6.13 × 10^−4^ S cm^−1^) and *t*_Li_^+^ (0.63) at RT, while the crystal volume of PEGDMA 1000 decreased drastically after in situ UV-curing polymerization. Furthermore, the Li//LFP cell assembled with the prepared PE provides excellent cyclic stability and rate performance. This research work aims to develop a modification method to improve the feasibility of PEO-based polymer electrolytes in LIBs.

## 2. Experiments

### 2.1. Materials

Poly(ethylene glycol) 1000 (PEG 1000, average M.W.: 950–1050), triethylamine (99%) sodium chloride (99%), sodium bicarbonate (99.5~100.5%) and magnesium sulfate anhydrous (99.5%) were purchased from Samchun Chemical (Seoul, South Korea). 2-hydroxy-2-methylpropiophenone (HMPP, 97%), methacryloyl chloride (97%) and LiFSI (ultradry, 99.9%) were supplied by Sigma-Aldrich (Seoul, South Korea). Solvents include dichloromethane (DMC) and DMF from Alfa Aesar and they were distilled for purification prior to use. LFP single-side-coated aluminum foil and lithium foil were purchased from MTI Corporation (Richmond, CA, USA) and vacuum heated at 100 °C before in situ polymerization.

### 2.2. Instrumentations and Measurements

The monomer chemical structure was confirmed by proton nuclear magnetic resonance (^1^H-NMR) spectra on a JNM-ECZ500R/S1 (JEOL, Tokyo, Japan) at 400 MHz. Chloroform (CDCl_3_) and tetramethylsilane (TMS) are solvents and internal standards. The polymerization was monitored by an infrared Fourier transform (FT-IR) using a Nicolet iS5 (Waltham, US) between 4000 and 500 cm^–1^ with a spectral resolution of 4 cm^–1^. The thermal stability of the electrolytes was evaluated on a TGA-8000 (Perkin Elmer, Waltham, MA, USA) by heating to 500 °C at a heating rate of 10 °C min^−1^ at a nitrogen flow rate of 50 mL min^–1^. Differential scanning calorimetry (DSC) was measured by Perkin-Elmer DSC 6000 (Waltham, MA, USA) in a temperature range from −60 to 300 °C with a 10 °C min^−1^ heating/cooling rate under a nitrogen atmosphere. Field emission scanning electron microscopy (FE-SEM) and energy-dispersive X-ray spectroscopy (EDS) analyses were performed on a JSM-6700F (JEOL, Japan) with an accelerating voltage of 15.0 kV to investigate the surface morphology and elemental composition of materials. X-ray diffraction analysis (XRD) was recorded on D2 Phaser (Bruker, Germany) from 10° to 80° at a speed rate of 2° min^−1^ at RT. Herein, the samples for characteristics and physiochemical measurement were prepared in a vial as shown in Figure S2b of the Supporting Information (SI). The electrochemical properties of the polymer electrolytes were calculated on the basis of electrochemical impedance spectroscopy (EIS) on the IM6ex, Zahner-Elektrik GmbH & Co. KG instrument (Kronach, Germany). The in situ LFP-PEs were sandwiched between two stainless-steel blocking electrodes of 15.8 mm diameter. The EIS was recorded in a temperature range of 30–80 °C and a frequency range from 0.1 Hz to 1 MHz with an alternating current (AC) amplitude of 10 mV. Z-view (version 3.1, Scribner Associates Inc., Southern Pines, NC, USA) was utilized to fit the EIS spectra to the corresponding equivalent circuit model. All electrochemical stability and cell performance of the prepared polymer electrolytes were measured on an Ivium-n-Stat (Ivium Technologies B.V., Eindhoven, The Netherlands) at RT.

### 2.3. Synthesis of PEGDMA 1000 

**Scheme 1:**
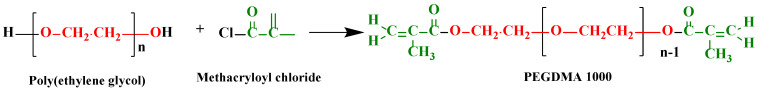
Schematic reaction for the synthesis of PEGDMA 1000.

The PEGDMA monomer was synthesized from PEG with methacryloyl chloride by an esterification reaction (Scheme 1). PEG 1000 (4.0 g, 4 mmol) was first dissolved in DMC (10 mL) in a 100 mL 2-necked flask equipped with a dropping funnel, condenser and magnetic stirrer. Then, triethylamine (1.69 mL, 12 mmol) was added to the solution as a catalyst and stirred for 30 min at RT. Methacryloyl chloride (0.78 mL, 8.0 mmol) with DMC (10 mL) was added dropwise to the mixture under ice conditions and stirred for 17 h under a nitrogen atmosphere. After the esterification reaction was complete, the mixture was poured into a supersaturated sodium bicarbonate solution and washed several times with a supersaturated sodium bicarbonate solution, sodium chloride solution and deionized water in order to remove residual methacryloyl chloride. The raw product solution was dried with anhydrous magnesium sulfate and the filtrate was distilled under reduced pressure at 40 °C. Finally, 4.1 g PEGDMA 1000 was obtained with 89% yield and stored under cool conditions.

### 2.4. Preparation of PEs on the LFP Cathode and Half-Cell

The synthesized PEGDMA 1000 and various weight ratios of LiFSI and DMF (10 wt.% of LiFSI) were mixed in a 10 mL vial. HMPP (1 wt.% of PEGDMA 1000) was added as a photoinitiator and stirred at RT for 1 h to obtain a clear and homogeneous electrolyte precursor solution. The electrolyte solution was then dripped onto the LFP cathode (d = 12 mm) and kept at RT for 12 h in order to sufficiently infiltrate the electrode. An electrolyte-coated LFP cathode (LFP-PEs) was exposed under an Hg UV lamp for 10 min to polymerize PEGDMA 1000 in situ. The thickness of LFP-PEs was controlled between 300 and 450 µm. The obtained electrolytes were labeled LFP-PE10, LFP-PE30 and LFP-PE50, respectively, where the numerical suffix represents the weight ratio of LiFSI to PEGDMA 1000. The half-cell with LFP-PEs//Li structure was assembled in an argon glove box (H_2_O and O_2_ < 1 ppm at RT) and aged at RT for 24 h before measurement. Scheme S1 describes the preparation details of both LFP-PEs and half-cells. For comparison, the polymer electrolyte without DMF was named LFP-SPEs and obtained by the same approach as LFP-PEs. Besides, an ex situ PE was fabricated on a glass plate and consisted of the same material ratio as in situ PEs. 

## 3. Results and Discussion

### 3.1. Characteristics and Physiochemical Properties of PEGDMA 1000 and Polymer Electrolytes

**Figure 1:**
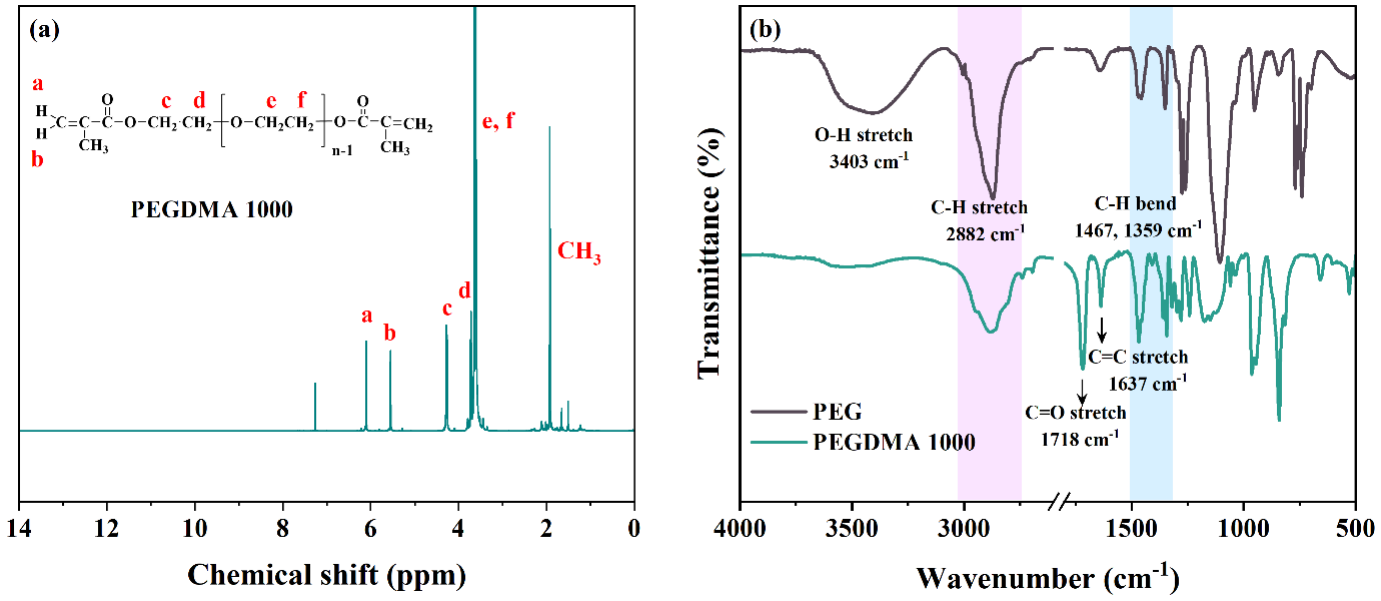
(**a**) ^1^H-NMR spectra of PEGDMA 1000, (**b**) FT-IR spectra of PEGDMA 1000.

Figure 1a and Figure S1 present the ^1^H-NMR results of the synthesized PEGDMA 1000, PEG and methacryloyl chloride. The CH_2_ and methyl protons from methacryloyl chloride are observed at 6.50, 6.03 and 2.01 ppm. The characteristic proton in the PEG backbone (O–CH_2_-CH_2_–O) and -OH proton peaks show signals at about 3.61 and 2.70 ppm (Figure S1). After esterification, the chemical shifts assigned to the PEGDMA CH_2_ chain are displayed at approximately 4.3 and 3.8 ppm and the vinyl and methyl protons of PEGDMA are at 6.11, 5.55 and 1.92 ppm, respectively. Meanwhile, the OH chemical shift in PEG disappeared from the NMR spectra after the esterification reaction, indicating complete synthesis of PEGDMA 1000. Moreover, speculation about the PEGDMA 1000 structure is further supported by FT-IR. In Figure 1b, the intense absorption at 1718 and 1637 cm^−1^ is attributed to C=O and C=C stretching of PEGDMA 1000 and also the CH_3_ stretching can be found at 2882 cm^−1^. On the contrary, no terminal OH stretching of PEG is observed in the region of 3403 cm^−1^, suggesting successful synthesis of PEGDMA 1000 [49,50]. 

The measured ^1^H-NMR data and FT-IR of PEGDMA 1000 were analyzed and presented as follows: ^1^H-NMR (400 MHz, CDCl_3_, δ): 1.92 (s, 3H, C–CH_3_), 3.62 (s, O–CH_2_–CH_2_–O), 3.71–3.73 (m, 2H, O–C–CH_2_–O), 4.26–4.29 (m, 2H, O–CH_2_–C–O), 5.54–5.55 (s, 1H, C*H*H=C) and 6.10–6.11 (s, 1H, CH*H*=C) ppm. FT-IR (KBr): 1467, 1359 (C-H bending), 1637 (C=C stretching), 1718 (C=O stretching) and 2882 (C-H stretching) cm^−1^. 

**Figure 2:**
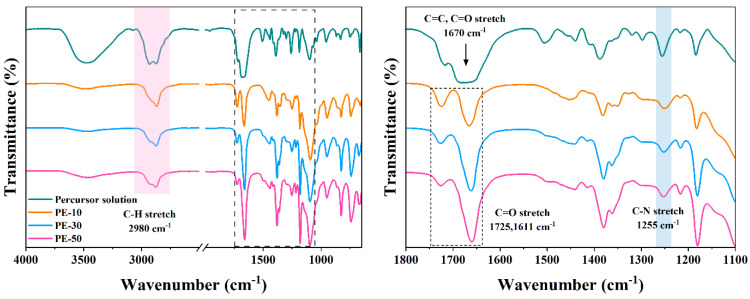
FT-IR spectra of PEs’ precursor solution and after UV-curing polymerization and enlarged graph (from 1800 to 1100 cm^−1^ wavenumber).

Figure S2 macroscopically depicts the electrolyte photos before and after UV-curing photopolymerization. The precursor electrolyte solution mixed with PEGDMA 1000, LiFSI and DMF is transportable and viscous, which turns into a soft and elastic polymer electrolyte after polymerization. In this work, LiFSI was employed as a lithium salt to design a polymer electrolyte, which is considered a promising candidate for commercial LiPF_6_ due to its higher σ, thermal stability and lithium-ion transference number [51]. The large-sized imide anion of sulfonyl imide groups (-SO_2_-N-SO_2_-) in LiFSI easily dissociates in the PEG matrix and reduces the crystalline phase of PEG. Therefore, LiFSI-PEG tends to improve ion transport, thereby increasing the electrochemical stability [52]. Besides, DMF worked as an additive in the electrolytes, so the amount was fixed at 10 wt.% of LiFSI. FT-IR further confirmed the electrolyte polymerization before/after UV curing. As shown in Figure 2, the broad absorption peak at ca. 1670 cm^−1^ is attributed to the C=C and C=O stretching of PEGDMA 1000 and DMF and the characteristic C=O stretching vibration of PEGDMA 1000 occurs at ca. 1725 cm^−1^ [53]. After UV irradiation, the broad absorption peak is dramatically narrowed due to the disappearance of the C=C stretching of PEGDMA 1000 and the C=O stretching absorption peak of PEGDMA 1000 and DMF is maintained at 1725 and 1661 cm^−1^ in the spectra, respectively [54]. Such changes in chemical structure proved the successful photopolymerization of PEGDMA 1000, LiFSI and DMF. 

FT-IR (KBr): 2980 (C-H stretching), 1725 (C=O stretching of PEGDMA 1000), 1670 (C=C stretching of PEGDMA 1000 and C=O stretching of DMF), 1661 (C=O stretching of DMF) and 1255 (C-N stretching of DMF) cm^−1^.

**Figure 3:**
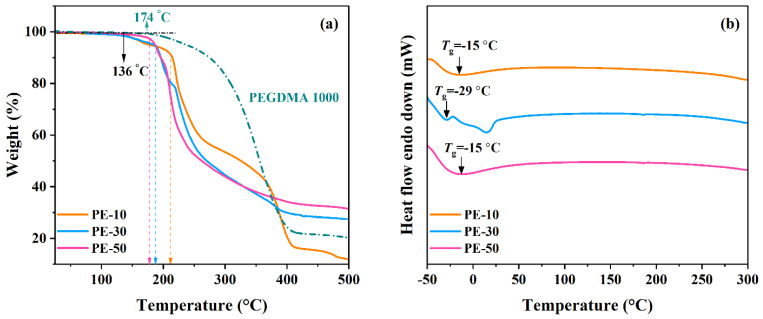
(**a**) TGA and (**b**) DSC curves of PEGDMA 1000 and PEs.

TGA and DSC were utilized to study the thermal properties of monomers and electrolytes. The weight degradation curves of PEGDMA 1000 and electrolytes from 30 to 500 °C are illustrated in Figure 3a. The synthesized PEGDMA 1000 exhibits thermal stability up to ca. 174 °C. The produced electrolytes show a two-step weight decomposition; the first weight degradation at ca. 136 °C is accounted for by DMF weight loss; the second weight loss is observed from 178, 187 and 213 °C for PE-10, PE-30 and PE-50, which were assigned to the FSI^-^ group and the main PEGDMA chain, which is significantly superior to the commercial liquid electrolyte [53,55]. 

According to the Vogel–Tammann–Fulcher equation, high σ is obtained from the low glass transition temperature (*T_g_*) in the materials. Thus, the *T_g_* of the prepared polymer electrolytes was confirmed by DSC in a temperature range of −60–300 °C. In Figure 3b, all electrolytes present one *T_g_* endo peak before 300 °C, suggesting that amorphous electrolytes were fabricated after UV polymerization. Furthermore, the *T_g_* values for PE-10, PE-30 and PE-50 are observed at −15, −29 and −15 °C, respectively, which indicates that all prepared polymer electrolytes allow the chain to be highly flexible at RT for easy lithium-ion transport in the polymer matrix. The TGA and DSC results illustrate that all prepared electrolytes can offer promising thermal stability between −15 and 130 °C, which is satisfactory at the practical use temperature of the LIBs. 

**Figure 4:**
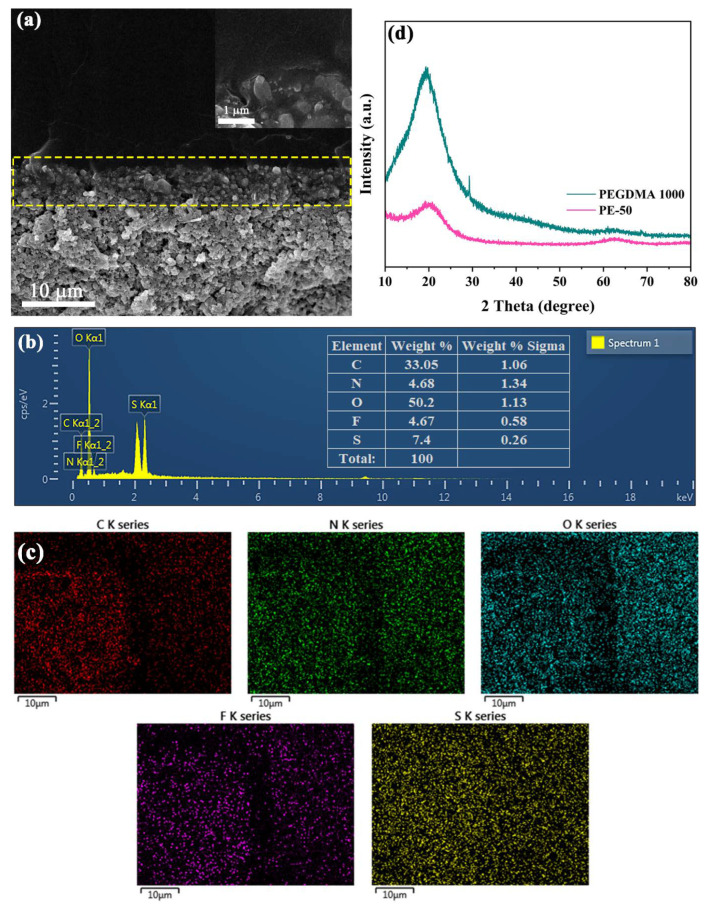
(**a**) Cross-sectional and (**b**) EDS analysis, (**c**) elemental mapping of C, N, O, F and S of PE-50, (**d**) XRD pattern of precursor solution and PE-50.

FE-SEM was utilized to observe the morphological characteristics of the LFP cathode and polymer electrolytes. The LFP-PE50 cross-sectional morphology image shown in Figure 4a indicates that the polymer electrolyte is completely connected with the LFP cathode. In addition, the fuscous area (yellow rectangle) on the LFP proves that the precursor electrolyte solution can permeate into the LFP cathode. Such promising incorporation further indicates that the PEs formed via in situ polymerization can effectively reduce the interfacial resistance between the electrodes and the polymer electrolytes. Additionally, the elemental composition of LFP-PE50 was analyzed by EDS and the results are presented in Figure 4b,c. As shown in Figure 4b, the PE-50 consists of C, O, N, F and S elements and the mapping images (Figure 4c) suggest that a uniform electrolyte layer was fabricated on the LFP cathode.

To verify the crystallographic phase of the PEGDMA 1000 and PE-50, XRD was conducted from 10° to 80° and the resulting patterns are displayed in Figure 4d. PEGDMA 1000 shows a high broad and shaped peak at 20° and 30°, indicating the semi-crystalline phase of the synthesized PEGDMA 1000. Moreover, the diffraction peak of PE-50 at ca. 20 drops dramatically after in situ polymerization, but the peak angle of both materials is about the same. Moreover, no other sharp peak was observed in the measuring range, indicating the amorphous phase of PE-50, which also corresponded to the results of the DSC test.

### 3.2. Electrochemical Properties of PEs and Coin Cells

**Figure 5:**
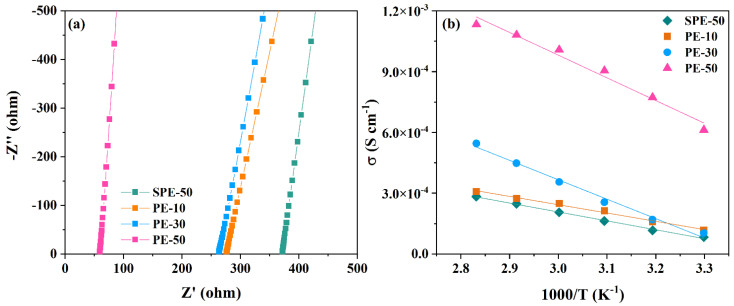
(**a**) Comparison of Nyquist plots of PEs at RT and (**b**) ionic conductivity versus temperature plots of PEs.

For further temperature-dependent σ study of PEs, EIS was collected from RT to 80 °C and fitted with an equivalent circuit model in Z-view software (Figures S3 and S4, Supporting Information). The σ was calculated using the equation σ = L/*R_b_* A, where L, *R_b_*, and A denote the thickness, the PE bulk resistance value and the effective contact area, respectively. The compared Nyquist plots and the σ results of PEs are described in Figure 5. It is apparent from Figure 5a that *R_b_* gradually decreases with increasing LiFSI content, suggesting an increase in enhanced Li-ion intercalation and deintercalation of ions in electrolytes. In contrast, the DMF-free electrolyte shows the highest *R_b_* value of any prepared electrolyte, reflecting the more difficult ion transfer and lower electrochemical kinetic performance in the polymer host. 

Figure 5b displays the calculated σ versus temperature plots for four prepared polymer electrolytes. All values of σ increase with increasing temperature in the RT–80 °C range and σ of PE-50 reaches 1.01 × 10^−3^ S cm^−1^ at 60 °C because of the increased polymer chain flexibility with temperature rise, which promotes facile ions’ transport in the polymer electrolyte [28,29]. Moreover, σ reaches 1.19 × 10^−4^ S cm^−1^, 1.24 × 10^−4^ S cm^−1^ and 6.13 × 10^−4^ S cm^−1^ at RT for PE-10, PE-30 and PE-50, respectively. However, the electrolyte without DMF presents the lowest RT σ value (8.35 × 10^−5^ S cm^−1^) in all investigated samples, suggesting that the addition of DMF was beneficial for ion transport in the polymer matrix. Furthermore, in our previous report, it was proposed that the actual σ value of a PEO-based electrolyte is affected by the lithium concentration, anionic nature, temperature, etc., and excess lithium concentration could lower the segmental mobility of PEO, leading to a low σ [56]. Herein, PE-50 reveals the best ion-transport properties and is almost an order of magnitude higher than the electrolyte without DMF over the entire temperature range studied, suggesting that the addition of DMF could give a satisfactory σ value even at a high lithium concentration. This result can probably be explained by the fact that DMF carbonyl groups interact with Li cations to provide much higher donor numbers, even under lithium-salt-rich conditions, which is beneficial to reduce cross-linking between lithium ions and ether oxygen and maintaining PEO segmental mobility [39,57]. 

**Figure 6:**
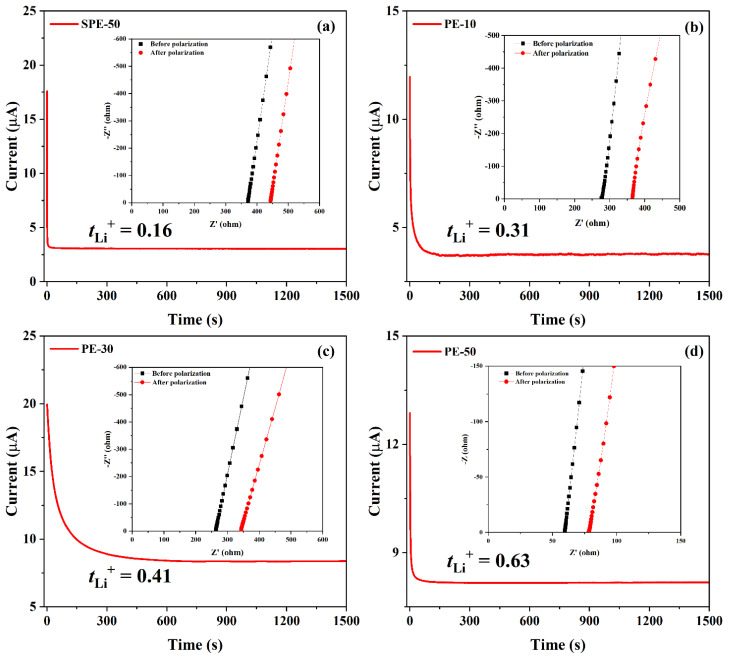
The chronoamperometry curve of the symmetrical cell Li/ PEs /Li at a polarization voltage of 10 mV. The inset shows the Nyquist plots of the cell before and after polarization for (**a**) SPE-50, (**b**) PE-10, (**c**) PE-30 and (**d**) PE-50, respectively, at RT.

The lithium *t*_Li_^+^ was calculated from the Bruce–Vincent equation using AC impedance spectroscopy combined with DC polarization at RT (Table S1). Figure 6 demonstrates the chronoamperometry profile and the AC impedance before and after polarization under a voltage of 10 mV. The calculated values of *t*_Li_^+^ are 0.31, 0.41 and 0.63 for PE-10, PE-30 and PE-50, which are at least twice the SPE-50 (0.16). σ and *t*_Li_^+^ results reflect the fabricated PEs, which can fully satisfy the practical application of LIBs at RT.

**Figure 7:**
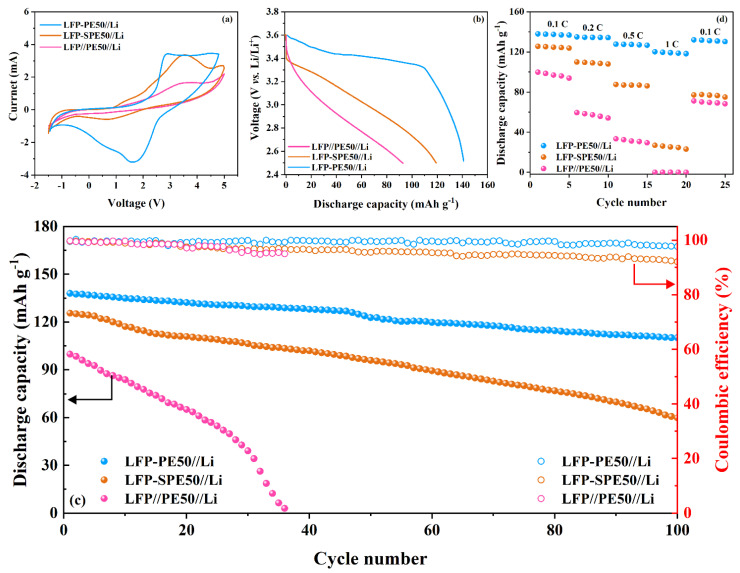
(**a**) CV plots of compared polymer electrolytes at room temperature with a scan rate of 1 mV s^−1^, (**b**) CD plots of PE-50, (**c**) cycling performance of half-cell at 0.1 C and (**d**) rate capability.

The electrolytes with 50 wt.% of LiFSI were used to further investigate cell performances because PE-50 showed the highest σ and *t*_Li_^+^ in this work. These half-cells (LFP//PE50//Li, LFP-SPE50//Li and LFP-PE50//Li) were assembled and compared to further estimate the in situ polymerization and the DMF effect on the electrolyte in electrochemical properties. Figure 7a describes a comparison of the first three cells’ cyclic voltammogram (CV) curves with a potential window from −1 to 5 V (vs. Li/Li^+^) at a scan rate of 1 mV s^−1^ at RT. Lithium foil was applied as both the counter and reference electrodes and LFP was used as a working electrode. As shown in the figure, there is a small oxidation peak and no apparent reduction peak for the LFP//PE50//Li cell, indicating a coin cell with an inadequate redox system. In the case of LFP-SPE50//Li, the obvious redox peaks assigned to lithium plating and stripping are in a region of 0.25 and 3.5 V, but the cell still showed poor reversibility in the investigated range (Figure S5). On the contrary, the LFP-PE50//Li cell exhibits the anodic and cathodic peaks at 1.75 and 2.75 V and almost overlapping CV curves, reflecting the promising cell electrochemical reversibility. To further study the particle application of fabricated PEs, a test of cell charging and discharging between 2.5 and 3.6 V (vs. Li/Li^+^) at RT under 0.1 C was performed. The C rate was defined based on the active LFP weight (about 12 mg cm^−2^). Figure 7b outlines the initial charging/discharging profile of the LFP//PE50//Li, LFP-SPE50//Li and LFP-PE50//Li cells. The obtained curves demonstrate that the LFP-PE50//Li cell offered a better discharge capacity of 141 mAh g^−1^ than the LFP//PE50//Li (96 mAh g^−1^) and LFP-SPE50//Li (119 mAh g^−1^) cells. Figure 7c shows the cycle performance for three coin cells at 0.1 C. The discharge capacity after 100 cycles for LFP-PE50//Li cell remains at 110 mAh g^−1^ (78.01% capacity retention), while LFP-SPE50//Li cells drop to 58 mAh g^−1^ (48.74 % capacity retention). On the contrary, the capacity of LFP//PE-50//Li cell plummets to 3 mAh g^−1^ after only 36 cycles, indicating a poor charging and discharging ability. Moreover, the observed Coulombic efficiency after 100 cycles for the LFP-PE50//Li cell remains at 97.6%, which is significantly above the value of 82.1% for the LFP-SPE50//Li cell.

The capacity rate of the three cells presented in Figure 7d shows that LFP//PE50//Li and LFP-SPE50//Li cells decreased sharply with the growth rate and almost dropped to 0 mAh g^−1^ by 1 C at RT for LFP//PE50//Li cell. In contrast, the LFP-PE50//Li cell maintains its specific capacity at 137, 136, 127 and 119 mAh g^−1^ at 0.1 C, 0.2 C, 0.5 C and 1.0 C, respectively, which is much better than the above-mentioned cells, especially at a high rate. In a brief of cell investigations, LFP-PE50//Li cells present superior electrochemical properties during charging/discharging, rate capacity and cycle test. This can be attributed to the in situ polymerization, considerably dropping interfacial resistance, and the polarization between LFP and PE-50. Moreover, the DMF in the PE fabricated flexible polymer chains, which is beneficial for cell stability. 

## 4. Conclusions

In conclusion, we synthesized a low-molecular-weight monomer, PEGDMA 1000, for use as a polymer matrix and combined it with DMF to fabricate a polymer electrolyte for LIBs. Herein, the PE-50 offered a high σ of 6.13 × 10^−4^ S cm^-1^ and a *t*_Li_^+^ of 0.63 at RT. Such an electrolyte was prepared by in situ polymerization and directly coated on the LFP cathode to create close contact with each other, which dramatically reduced the interfacial resistance between the electrolyte and the electrode. As a result, the LFP-PE50//Li cell showed good thermal stability up to 136 °C and a promising initial discharging capacity of 141 mAh g^−1^ and a retention capacity (78.01%) after 100 cycles at 0.1 C. Moreover, Coulombic efficiency and cycle performance were improved in the system cell. These results indicate the LFP-PE designed in this work can develop the practical application of PEO-based polymer electrolytes in LIBs. 

## Supplementary Materials

The following supporting information can be downloaded at: https://www.mdpi.com/article/10.3390/polym14173443/s1, Figure S1. (a) ^1^H-NMR spectra of methacryloyl chloride and (b) PEG. Figure S2. The macroscopical images of PE (a) precursor solution and (b) after UV-cured polymerization. Scheme S1. Schematic illustration of the (a) LFP-50 and (b) half-cell preparation. Figure S3. Impedance spectra of (a) SPE-50, (b) PE-10, (C) PE-30 and (d) PE-50. Figure S4. Z-view fitting result and current model. Table S1 Currents and resistances of the electrolytes for *t*_Li_^+^ calculation. Figure S5. CV plots of polymer electrolytes at room temperature with a scan rate of 1 mV s^−1^.
