# Supplementary material for: Improving the Ionic Conductivity of PEGDMA-Based Polymer Electrolytes by Reducing the Interfacial Resistance for LIBs
Source: Polymers (Basel). 2022 Aug 23;14(17):3443. doi: 10.3390/polym14173443 (PMC9460516; doi:10.3390/polym14173443)
Supplement: Supplementary file 1 [file polymers-14-03443-s001.zip › polymers-1871968-supplementary.pdf]

Article

Improving the ionic conductivity of PEGDMA-based polymer electrolytes by reducing the interfacial resistance for LIBs

Figure S1. (a) ^{1}H-NMR spectra of methacryloyl chloride and (b) PEG.
Figure S2. The macroscopical images of PE (a) precursor solution and (b) after UV-cured polymerization.
Scheme S1. Schematic illustration of the (a) LFP-50 and (b) half-cell preparation.
Figure S3. Impedance spectra of (a) SPE-50, (b) PE-10, (C) PE-30 and (d) PE-50.
Figure S4. Zview fitting result and current model.
Table S1. Currents and resistances of the electrolytes for t_{Li^+} calculation.
Figure S5. CV plots of polymer electrolytes at room temperature with a scan rate of 1 mV s^{-1}.

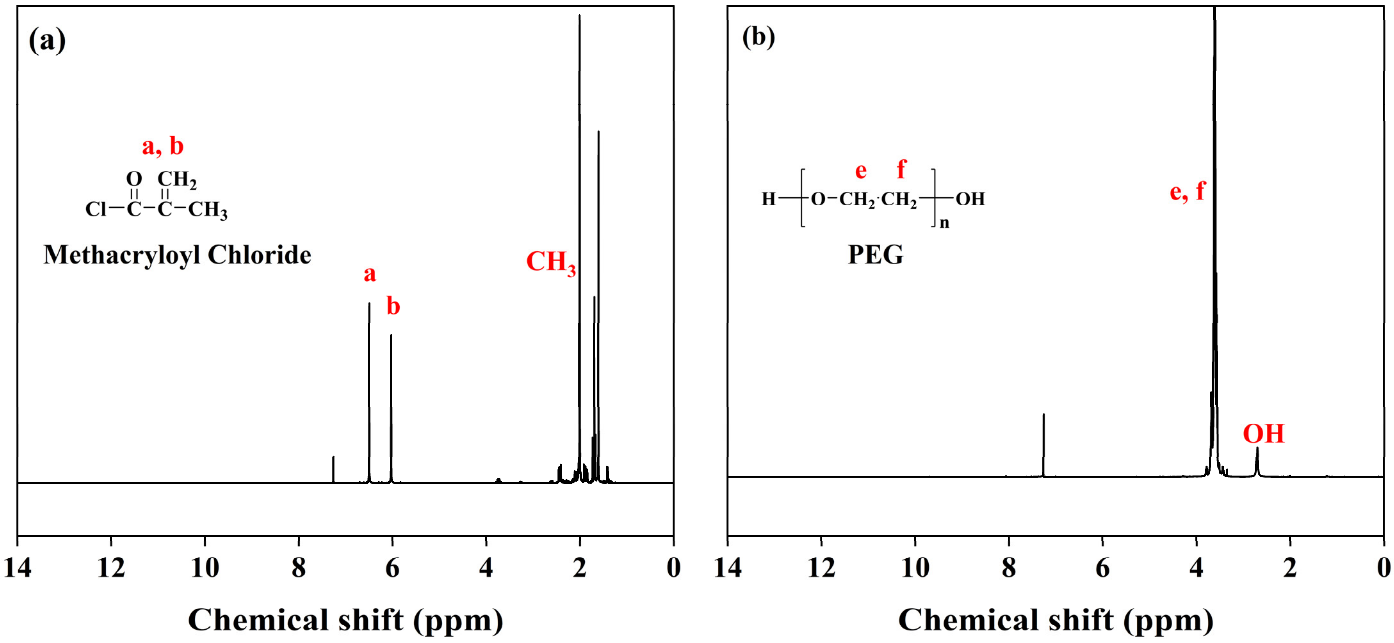

Figure S1. (a) ^{1}H-NMR spectra of methacryloyl chloride and (b) PEG.

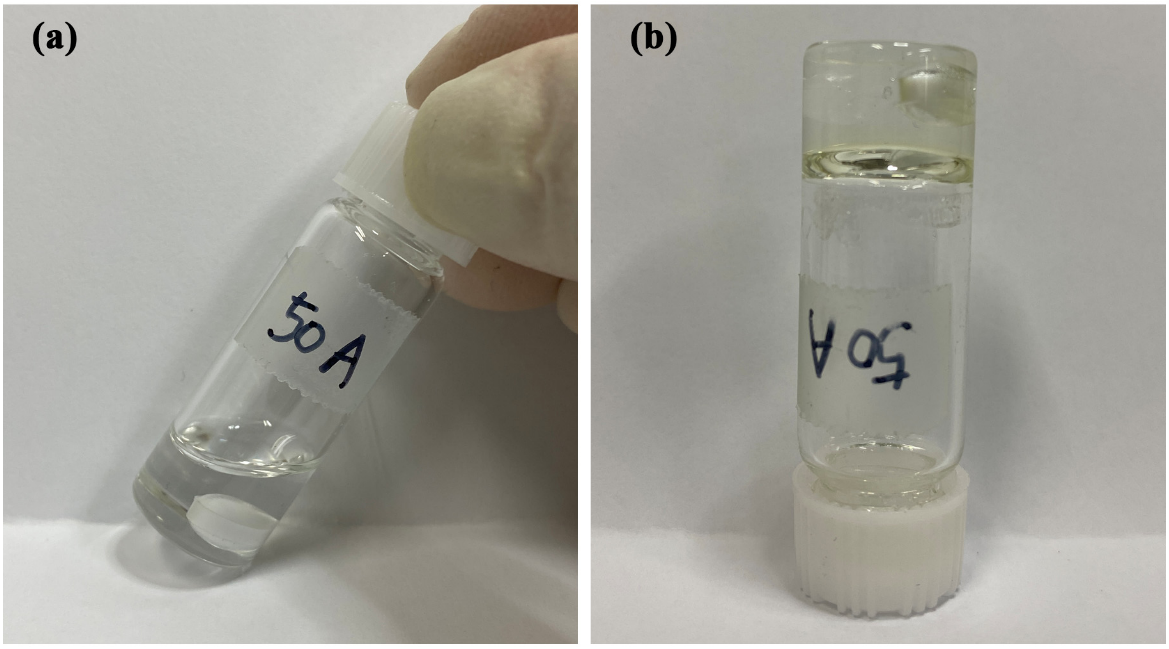

Figure S2. The macroscopical images of PE (a) precursor solution and (b) after UV-cured polymerization.

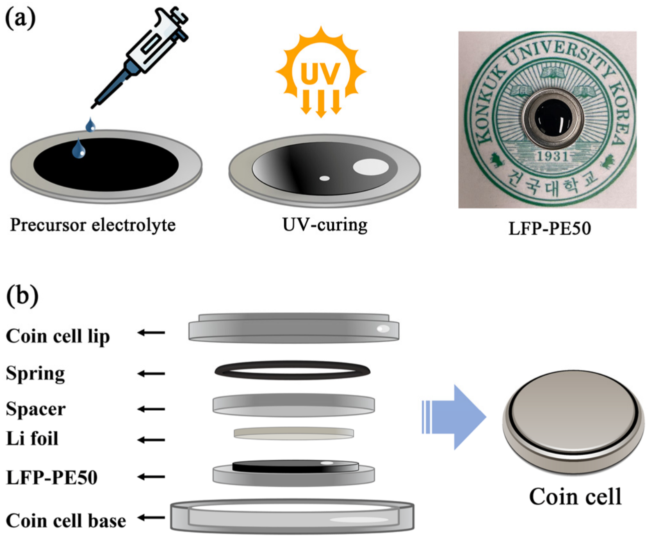

Scheme S1. Schematic illustration of the (a) LFP-50 and (b) half-cell preparation.

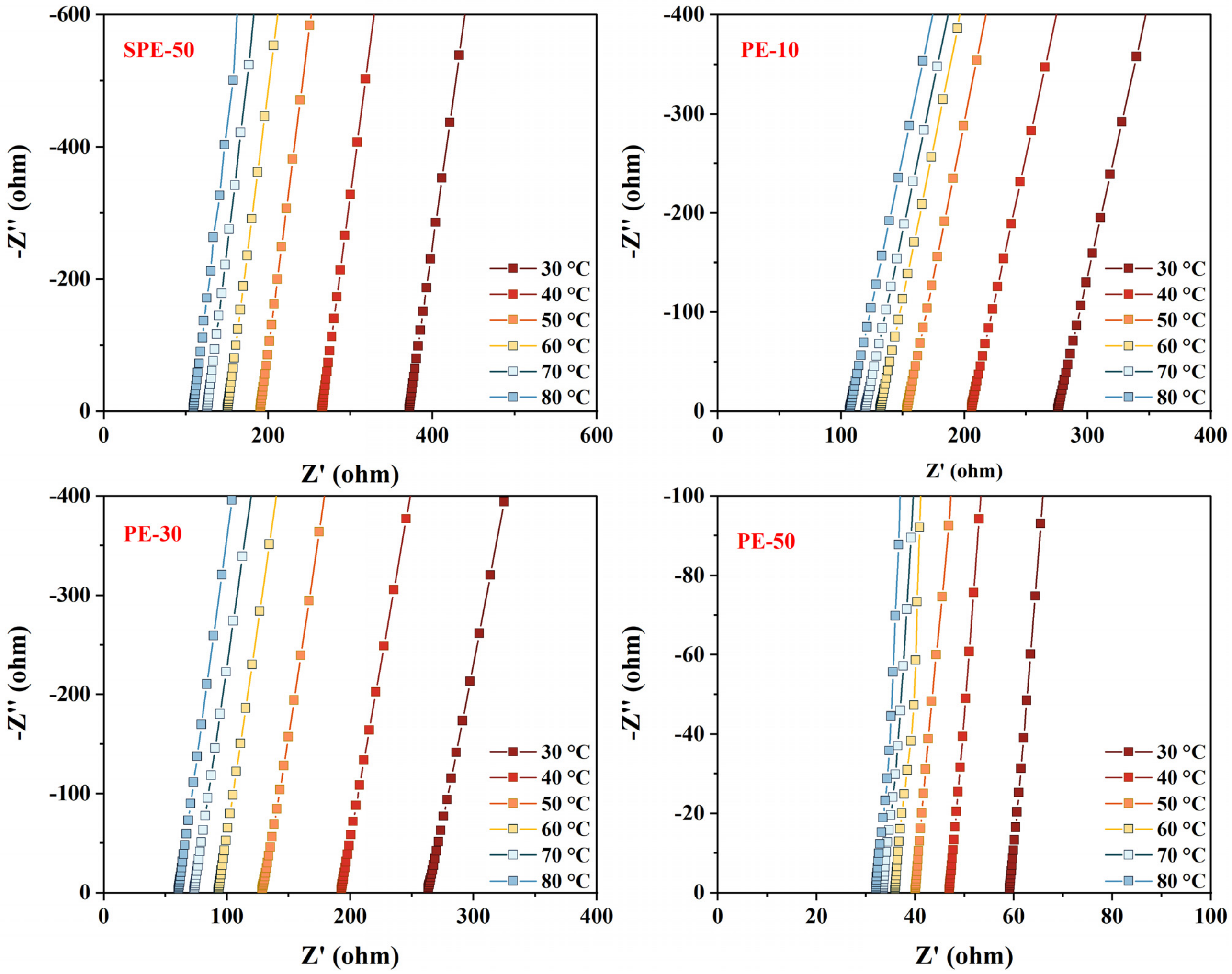

Figure S3. Impedance spectra of (a) SPE-50, (b) PE-10, (C) PE-30, and (d) PE-50.

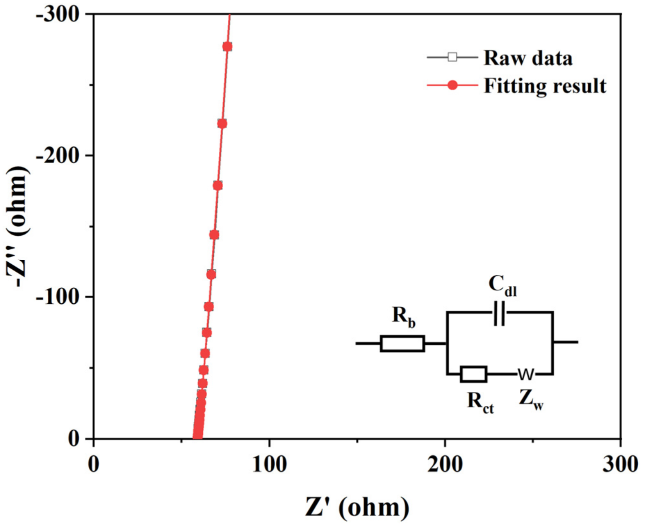

Figure S4. Zview fitting result and current model.

Whereas R_b is the bulk resistance represent the movement of lithium ions through the electrolyte. R_{ct}, C_{dl} and Z_w indicate the charge transfer resistance during the electrochemical reactions, double-layer capacitance and Warburg diffusion resistance, respectively.

Table S1. Currents and resistances of the electrolytes for t_{Li^+} calculation.

Electrolyte	Is (A)	I_0 (A)	ΔV (mV)	R_0 (ohm)	Rs (ohm)	t_{Li^+}
SPE-50	3.05E-06	1.76E-05	10	371	443	0.16
PE-10	3.77E-06	1.20E-05	10	276	364	0.31
PE-30	8.38E-06	2.00E-05	10	263	343	0.41
PE-50	8.18E-06	1.29E-05	10	59	79	0.63

Bruce–Vincent–Evans Equation:

$$t_{Li^+} = I_s (\Delta V - I_0R_0)/ I_0 (\Delta V - I_sR_s)$$

where I_0 and I_s are the initial and steady-state current, and R_0 and R_s are denoted as the interfacial resistance between electrode and electrolyte of the symmetrical cell before and after polarization, respectively.

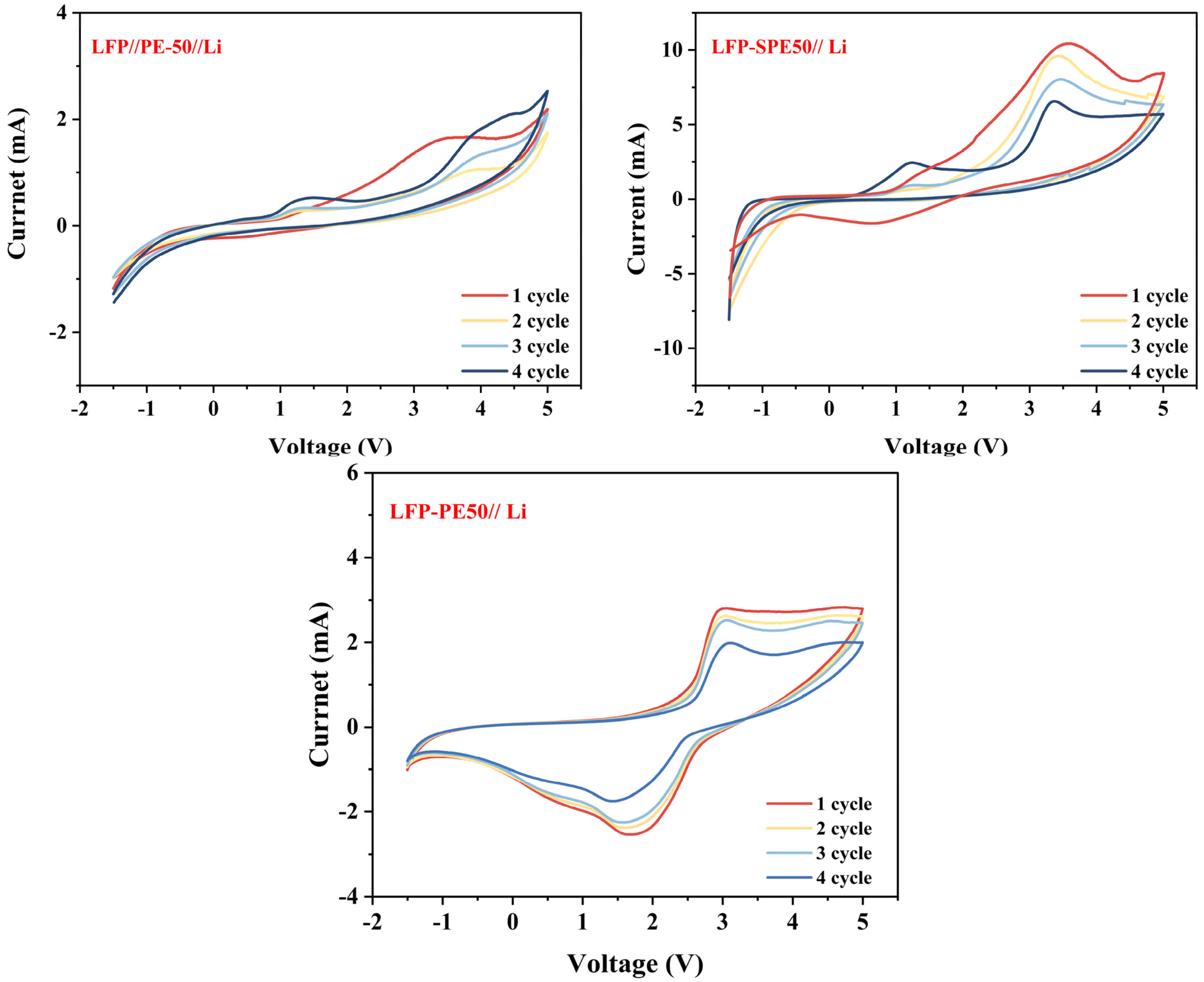

Figure S5. CV plots of polymer electrolytes at room temperature with a scan rate of 1 mV s^{-1}.
